# De Novo *FGF12* (Fibroblast Growth Factor 12) Functional Variation Is Potentially Associated With Idiopathic Ventricular Tachycardia

**DOI:** 10.1161/JAHA.117.006130

**Published:** 2017-08-03

**Authors:** Qianqian Li, Yuanyuan Zhao, Gang Wu, Shanshan Chen, Yingchao Zhou, Sisi Li, Mengchen Zhou, Qian Fan, Jielin Pu, Kui Hong, Xiang Cheng, Qing Kenneth Wang, Xin Tu

**Affiliations:** ^1^ Key Laboratory of Molecular Biophysics of the Ministry of Education College of Life Science and Technology Center for Human Genome Research Cardio‐X Institute Huazhong University of Science and Technology Wuhan China; ^2^ Key Laboratory for Molecular Diagnosis of Hubei Province The Central Hospital of Wuhan Tongji Medical College Huazhong University of Science and Technology Wuhan China; ^3^ Key Laboratory of Organ Transplantation Institute of Organ Transplantation Tongji Hospital Tongji Medical College Huazhong University of Science and Technology Ministry of Education and Ministry of Health Wuhan China; ^4^ Department of Cardiology Renmin Hospital of Wuhan University Wuhan China; ^5^ The Laboratory of Cardiovascular Immunology Institute of Cardiology Union Hospital Tongji Medical College of Huazhong University of Science and Technology Wuhan China; ^6^ State Key Laboratory of Cardiovascular Disease, Physiology and Pathophysiology Laboratory Fuwai Hospital National Center for Cardiovascular Diseases Chinese Academy of Medical Sciences and Peking Union Medical College Beijing China; ^7^ Department of Cardiovascular Medicine The Second Affiliated Hospital of Nanchang University and Jiangxi Key Laboratory of Molecular Medicine Jiangxi China; ^8^ Department of Molecular Cardiology Cleveland Clinic Cleveland Ohio USA

**Keywords:** fibroblast growth factor 12, genetic risk factor, variations, ventricular tachycardia, Arrhythmias, Genetic, Association Studies

## Abstract

**Background:**

Idiopathic ventricular tachycardia (VT) is a type of cardiac arrhythmia occurring in structurally normal hearts. The heritability of idiopathic VT remains to be clarified, and numerous genetic factors responsible for development of idiopathic VT are as yet unclear. Variations in *FGF12* (fibroblast growth factor 12), which is expressed in the human ventricle and modulates the cardiac Na^+^ channel Na_V_1.5, may play an important role in the genetic pathogenesis of VT.

**Methods and Results:**

We tested the hypothesis that genetic variations in *FGF12* are associated with VT in 2 independent Chinese cohorts and resequenced all the exons and exon–intron boundaries and the 5′ and 3′ untranslated regions of *FGF12* in 320 unrelated participants with idiopathic VT. For population‐based case–control association studies, we chose 3 single‐nucleotide polymorphisms—rs1460922, rs4687326, and rs2686464—which included all the exons of *FGF12*. The results showed that the single‐nucleotide polymorphism rs1460922 in *FGF12* was significantly associated with VT after adjusting for covariates of sex and age in 2 independent Chinese populations: adjusted *P*=0.015 (odds ratio: 1.54 [95% CI, 1.09–2.19]) in the discovery sample, adjusted *P*=0.018 (odds ratio: 1.64 [95% CI, 1.09–2.48]) in the replication sample, and adjusted *P*=2.52E‐04 (odds ratio: 1.59 [95% CI, 1.24–2.03]) in the combined sample. After resequencing all amino acid coding regions and untranslated regions of *FGF12*, 5 rare variations were identified. The result of western blotting revealed that a de novo functional variation, p.P211Q (1.84% of 163 patients with right ventricular outflow tract VT), could downregulate *FGF12* expression significantly.

**Conclusions:**

In this study, we observed that rs1460922 of *FGF12* was significantly associated with VT and identified that a de novo variation of *FGF12* may be an important genetic risk factor for the pathogenesis of VT.

**Clinical Perspective**

**What Is New?**

- Our studies are the first analysis of the genetic association between *FGF12* (fibroblast growth factor 12) and ventricular tachycardia. The results revealed that a functional variation (p.P211Q) significantly reduced *FGF12* expression, which may affect the interaction of *FGF12* and Na^+^ channels and confer risk of right ventricular outflow tract ventricular tachycardia.

**What Are the Clinical Implications?**

- The findings suggest that known of variations of *FGF12* may help identify patients at risk of right ventricular outflow tract ventricular tachycardia in the population, stratify idiopathic ventricular tachycardia, and develop a novel potential treatment.

## Introduction

Idiopathic ventricular tachycardia (VT) is a distinct type of monomorphic VT that occurs commonly without structural heart disease.^1^ The morbidity of idopathic VT is ≈10% in the United States and 20% in Japan.^2^ Genetic factors play an important role in the pathogenesis of idopathic VT.^3^ Mutations in genes such as *RYR2* (Ryanodine receptor 2), *DPP6* (Dipeptidyl aminopeptidase‐like protein 6), *CASQ2* (Calsequestrin‐2), *TRDN* (Triadin), *CALM1* (Calmodulin‐1), *SCN5A* (Sodium channel protein type 5 subunit alpha), *SCN4B* (Sodium channel subunit beta‐4), *KCNQ1* (Potassium voltage‐gated channel subfamily KQT member 1), *KCNE1* (Potassium voltage‐gated channel subfamily E member 1), *KCNJ2* (Inward rectifier potassium channel 2), *KCNH2* (Potassium voltage‐gated channel subfamily H member 2), *KCNJ5* (Inward rectifier potassium channel 2), *KCNJ8* (ATP‐sensitive inward rectifier potassium channel 8), *KCNE2* (Potassium voltage‐gated channel subfamily E member 2), *CACNB2* (Voltage‐dependent L‐type calcium channel subunit beta‐2), *CACNA1C* (Voltage‐dependent L‐type calcium channel subunit alpha‐1C), and *CACNA2D1* (Voltage‐dependent calcium channel subunit alpha‐2)^3^, ^4^, ^5^ have been identified as causes of inherited arrhythmogenic disorders, including idopathic VT.

Fibroblast growth factor (FGF) homologous factors (FHFs; FGF11–14) are members of FGFs. Different from other secretory FGFs (FGF1–10 and FGF15–23), FHFs belong to intracellular nonsecretory forms.^6^ FHFs lack signal sequence, cannot release from cells,^7^ and activate FGF receptors.^8^ FHFs can modulate both Na^+^ and Ca^2+^ channels, and genes encoding FHFs are responsible for the development of Brugada syndrome (BrS), characterized by VT or ventricular fibrillation without cardiac abnormality.^9^, ^10^, ^11^ Recent studies also demonstrated that a missense mutation (p.Q7R) in *FGF12* (encoding a member of FHFs that expresses abundantly in the human ventricle) is a disease‐associated functional variation of BrS. The p.Q7R mutation reduced binding to the Na_V_1.5 C‐terminus and Na^+^ channel current density, leading to Na^+^ channel loss‐of‐function phenotype consistent with that in BrS.^12^, ^13^ Furthermore, Musa et al reported that a variation in *SCN5A* (p.H1849R) blocks the regulation of FGF12 and causes human arrhythmia.^14^ This evidence highlighted that the variations in *FGF12* may affect the interaction between FGF12 and Na^+^ channel, leading to arrhythmia.

Because changes in sodium channel function are important in the pathogenesis of idopathic VT and other inherited arrhythmias, we supposed that variations in *FGF12* may be associated with VT/idopathic VT. To test the potential association between *FGF12* and VT/idopathic VT, we performed a 3‐stage study. In the first stage, we chose 3 single‐nucleotide polymorphisms (SNPs)—rs1460922, rs4687326, and rs2686464—that included all exons of *FGF12* to observe the association between *FGF12* and VT/idopathic VT in a Chinese population (case:control of 255:289). In the second stage, we replicated the result of the first stage in an independent sample (case:control of 180:288). In the third stage, we resequenced all the exons and exon–intron boundaries and the 5′ and 3′ untranslated regions (UTRs) of *FGF12* in 320 unrelated participants with idopathic VT to identify functional variations with risk effect on disease.^15^, ^16^

## Methods

### Study Samples

All participants were of Chinese descent and were chosen from GeneID.^17^, ^18^, ^19^, ^20^ The study was approved by appropriate local institutional review boards on human subject research and conformed to the guidelines set forth by the Declaration of Helsinki. Written informed consent was obtained from all participants.

A total of 255 participants with VT and 289 controls were enrolled in the first stage (discovery sample); 180 participants with VT and 288 controls were enrolled in the second stage (replication sample); 320 unrelated participants with idopathic VT (including 31 patients with idopathic VT from discovery and replication samples) were resequenced in the third stage.

All participants were precisely diagnosed with VT by ECG and/or Holter ECG recordings. VT was diagnosed according to the standards mentioned in the American College of Cardiology, American Heart Association, and European Society of Cardiology ventricular arrhythmia guidelines.^21^ Briefly, a patient showing wide‐QRS complex tachycardia on ECG was diagnosed as a patient with VT. Idopathic VT was defined as VT with structurally normal heart, and participants with coronary artery disease, ischemic stroke, congestive heart failure, essential hypertension, or diabetes mellitus were excluded.^2^ Those without a history of arrhythmia or detectable abnormal ECG were defined as controls. Demographic and other relevant clinical information, if present, was obtained from the medical records.

### SNP Selection and Genotyping

A total of 157 SNPs flanked the 266.3 kb genomic region of *FGF12* on chromosome 3 (International HapMap Project showed from 193 342 424 to 193 608 706 bp). Three SNPs—rs1460922, rs4687326, and rs2686464—were selected from the genotyped SNPs in the Han Chinese population of the HapMap project (the phase 2 database) using Haploview 4.2 for the study. The 3 SNPs were located in different linkage disequilibrium blocks and covered all exons and regulatory regions of *FGF12* (D′=1, *r* ^2^ between 0.048 and 0.5; Figures S1 and S2).

Human genomic DNA was extracted from the peripheral white blood cells using the Wizard Genomic DNA Purification Kit (Promega). The primer sequences are given in Table 1. All SNPs were genotyped by a Rotor‐Gene 6000 high‐resolution melt system (Corbett Life Science) using standard protocols with minor modifications. Reaction mixture and genotyping procedures were described previously.^22^ Three positive controls with genotypes of 3 SNPs and a negative control of ddH_2_O were included during each high‐resolution melt run. Twenty samples were randomly selected for direct Sanger sequencing to confirm the accuracy of genotyping.

**Table 1:** The Primers of Genotyping and Mutational Analysis for *FGF12B*

Exon	Forward	Reverse
FGF12‐exon01‐HRM	gccctgattaaaatgaaaattga	tgcaaacatttattaaccttttcct
FGF12‐exon02‐HRM	ccggcgtttatttttagcag	cgtgcctgtcagcaattcta
FGF12‐exon03‐HRM	ttttatggatgtgggcaattt	aggcaagacacacttggaaa
FGF12‐exon04‐1‐HRM	caagcggaaagagaaagagc	tgcgaagtagacgtttgcac
FGF12‐exon04‐2‐HRM	ttcttccccttccacttggt	cactctccgggcttctactg
FGF12‐exon05‐HRM	tttgcagaaccccagctca	ctgggccctacatttgatttg
FGF12‐exon06‐HRM	ggattatttattcaaaaggtcactg	gcctaacatgatggttactccat
FGF12‐exon07‐HRM	gacaatagttttgatcggctca	cctgcattgctcctgatttt
FGF12‐exon08‐1‐HRM	cagaggacatggatttcaagc	ggcggtacagtgtggaagaa
FGF12‐exon08‐2‐HRM	gtaccgccagcaagaatcag	gggtccaacaaagacagtcag
FGF12‐exon09‐1‐HRM	tgaaggaaatttatgtccactg	agggaagaaggggagagttc
FGF12‐exon09‐2‐HRM	tgagaactctccccttcttcc	ccactaggtcttgcgttgtc
rs1460922‐HRM	cacgtgcacaaagattagcac	ttcaattctccaaatcctttcc
rs4687326‐HRM	tgtatggtgccatattgtttcc	tgcagtttggtagattatcagc
rs2686464‐HRM	gggccagactctcttaacca	atcccactccgaagtccag
FGF12‐exon01‐SEQ	gggatgtgggctagctagatt	ggaaagtatatctccccttttgg

### *FGF12* Variation Analysis by Direct DNA Sequencing

All exons of *FGF12* were screened to find functional variations or alleles by polymerase chain reaction (PCR) and DNA Sanger sequencing in 320 unrelated patients with idopathic VT with the clear subtype of VT (including 31 patients with idopathic VT from the discovery and replication samples). The information on primers for PCR and sequencing are also shown in Table 1. Variations observed in *FGF12* were verified in 1000 control individuals chosen from our GeneID database. Those without a history of arrhythmia or detectable abnormal ECG were selected and randomly picked as controls.

### Bioinformatics Analysis for Variations in *FGF12*

To observe the possible function of rare variations in *FGF12*, conservative analysis of mutational amino acid was performed by the Center for Integrative Bioinformatics VU (http://www.ibi.vu.nl/programs/pralinewww/). ExAC Browser (http://exac.broadinstitute.org/) and MutationTaster (http://www.mutationtaster.org/) were used to test the frequency of the detected variants. The extent of injury of variations was predicted by Variant Effect Predictor online (http://www.ensembl.org/info/docs/tools/vep/index.html). SIFT (http://sift.jcvi.org/), PolyPhen2 (http://genetics.bwh.harvard.edu/pph2/), and Condel (https://omictools.com/consensus-deleteriousness-score-of-missense-snvs-tool) were used to predict the extent of injury. The possible transcriptional factor binding regions of noncoding variations discovered in the 5′ UTR of *FGF12* were predicted by using TFSEARCH (http://diyhpl.us/~bryan/irc/protocol-online/protocol-cache/TFSEARCH.html) and the JASPAR database (http://jaspar.genereg.net/cgi-bin/jaspar_db.pl?rm=browse&db=core&tax_group=vertebrates).

### Cell Lines and Plasmids

Rat myocardial H9C2 cells and Hela cells (human epitheloid cervix carcinoma cell) were purchased from the American Type Culture Collection. Cells were cultured in Dulbecco's modified Eagle's medium supplemented with 10% fetal bovine serum in a humidified incubator with 5% CO_2_ at 37°C.

We amplified the full‐length coding region of *FGF12* using human genomic cDNA, and it was subcloned into the p3xFLAG‐CMV‐10 by Tianyi Huiyuan. The plasmid of variant p.P211Q was constructed using PCR‐based site‐directed mutagenesis, and the construct was referred to as *p3xFLAG‐CMV‐10‐FGF12‐Mut*. Primers used for constructing p3xFLAG‐CMV‐10‐FGF12‐Mut were 5′‐GTATGTACAGAGAACAATCGCTACATGAAAT‐3′ (forward) and 5′‐ATTTCATGTAGCGATTGTTCTCTGTACATAC‐3′ (reverse). The promoter including the 5′ UTR of *FGF12B* was amplified by PCR using human genomic DNA as the template. The PCR product was digested with *Nhe*I and *Hin*dIII, and subcloned into the pGL3‐Basic luciferase plasmid, resulting in pGL3‐Basic‐5′‐UTR‐Wt. Two pairs of primers were used for constructing pGL3‐Basic‐5′‐UTR‐Wt. The first pair was 5′‐GGG ACT GAG TGA TCG GCC TTG CGT CCG GCG GGT AA‐3′ (forward) and 5′‐TGT TGG ACT CCC TCG CCT GCC GCT TCT G‐3′ (reverse), and the second pair was 5′‐GCC GCT AGC GCG GGT CAC TTC CTT CCT CGG CCG GGA TGG GCG GCG CGG G‐3′ (forward) and 5′‐GGC AAG CTT AGC TGC TCA GCG AGG GCC TCA GGC‐3′ (reverse). The plasmid of variant c.G723A was constructed as described, and the primers used were 5′‐TAG CAC TGC CTC CCC ACG ACT GCC CTT TCC C‐3′ (forward) and 5′‐GGG AAA GGG CAG TCG TGG GGA GGC AGT GCT A‐3′ (reverse).

### Dual Luciferase Reporter Assays

Hela cells were cultured in 24‐well plates for 24 hours and transfected with 250 ng pGL3‐Basic‐FGF12B‐5′UTR‐Wt or pGL3‐Basic‐FGF12B‐5′UTR‐Mut (c.723G>A), and 250 ng transcription factors (pCDNA3.1[+]‐MZF1 and p3xFLAG‐CMV‐10‐ZNF354C) or empty vector (pCDNA3.1[+] and p3xFLAG‐CMV‐10), along with 20 ng of the pRL‐TK vector containing the Renilla luciferase gene. Transfection was carried out using 1 μL lipofectamine 2000 and 500 μL Opti‐MEM (Gibco Life Technologies) reduced serum medium, according to the manufacturer's protocol. Cells were harvested 48 hours after transfection and lysed using 1× passive lysis buffer. Luciferase assays were performed as described in our previous study^23^ and using the Dual‐Glo luciferase assay kit (Gibco Life Technologies). The ratio of firefly over Renilla luciferase activities was calculated and considered as the final luciferase activity value. Each assay was performed in triplicate and repeated at least 3 times.

### Western Blot Analysis

H9C2 cells were cultured in 12‐well plates for 24 hours and transfected with 2 μg either p3xFLAG‐CMV‐10‐FGF12‐Wt or p3xFLAG‐CMV‐10‐FGF12‐p.P211Q, while empty vector (p3xFLAG‐CMV‐10) was used as a negative control. After 48 hours, transfected cells were collected and incubated in ice‐cold TNEN lysis buffer (50 mmol/L Tris/HCl, pH 7.5, 150 mmol/L NaCl, 2.0 mmol/L EDTA, 1.0% Nonidet P‐40) with 1 mini tab of EDTA‐free protease inhibitors and 1 mmol/L phenylmethylsulfonyl fluoride for 30 minutes at 4°C. The insoluble fraction was pelleted by centrifugation at 12 000*g* for 15 minutes at 4°C. Supernatant (100 μL) was mixed with 20 μL 6× Laemmli buffer (0.3 mol/L Tris‐HCl, 6% SDS, 60% glycerol, 120 mmol/L dithiothreitol, and proprietary pink tracking dye), and heated at 100°C for 10 minutes. Then, 40 μL samples were subjected to SDS‐PAGE (10%). After electrophoresis, proteins were transferred onto a 0.45‐μm polyvinylidene fluoride membrane. The membrane was probed with an anti‐DDDDK‐tag mouse monoclonal antibody (1:3000), followed by incubation with horseradish peroxidase–conjugated secondary goat antimouse antibody (1:5000). The protein signal was visualized by a Super Signal West Pico Chemiluminescent substrate (Pierce Chemical Co), according to the manufacturer's instructions. Human α‐tubulin (1:3000) was used as loading control. Each assay was performed in triplicate and repeated at least 3 times.

### Statistical Analysis

Power analysis of each study sample was conducted using the Power and Sample Size Calculations program (PS version 3.0.43, by William D. Dupont and Walton D. Plummer, Jr. http://ps-power-and-sample-size-calculation.software.informer.com/). The genotyping results of SNPs were screened for deviations from Hardy‐Weinberg equilibrium using PLINK version 1.07 (http://zzz.bwh.harvard.edu/plink/index.shtml), and no SNPs showed significant deviation (*P*>0.05). An independent *t* test was used to analyze the difference of sex and age in case and control groups by SPSS version 17.0 (IBM Corp). Association analysis for 3 SNPs before adjusting for covariates of age and sex were performed by 2×2 contingency tables using PLINK version 1.07. Association analysis for the 3 SNPs after adjusting for covariates of age and sex was performed using logistic regression analysis with SPSS version 17.0. Odds ratios (ORs) and corresponding 95% confidential intervals (CIs) were also calculated. In addition, we performed multiple logistic regression analysis to adjust significant covariates of sex and age for VT.

## Results

### Clinical Characteristics

The clinical characteristics of subjects with VT and controls are summarized in Table 2. Age and sex were also observed between cases and controls.

**Table 2:** Clinical Characteristics of Participants in This Study

Items	VT/VF Cases	Comparison Controls	*P*,* t* test
Discovery sample			
Sample size, n	255	289	
Sex, male, n (%)	157 (62)	194 (67)	*P*<0.001
Age, y, mean±SD	61±15	58±11	2.00E‐03
Replication sample			
Sample size, n	180	288	
Sex, male, n (%)	113 (73)	159 (55)	0.06
Age, y, mean±SD	47±18	62±8	*P*<0.001

VF indicates ventricular fibrillation; VT, ventricular tachycardia.

A total of 255 cases and 289 controls were enrolled in the discovery study. Among the cases, 62% were male, and the mean age was 61±15 years; among the controls, 67% were male, and the mean age was 58±11 years. A total of 180 cases and 288 controls were enrolled in the replication study. Among the study participants, 73% were male, and the mean age was 47±18 years; among the controls, 55% were male, and the mean age was 62±8 years. Statistical power analysis showed power >80% to detect the association between SNPs and VT in 2 samples and in the combined sample.

Among 320 unrelated patients with idopathic VT, 81 (25%) were male, and 239 (75%) were female. The mean age at diagnosis was 37±15 years. Among 163 (51%) cases with right ventricular outflow tract VT (RVOT), 113 (35%) cases showed left ventricular idopathic VT, 18 (6%) showed left ventricular outflow tract VT, and 26 (8%) showed miscellaneous VT.

### Significant Allelic Association Between SNP rs1460922 of *FGF12* and Risk of VT

The genotyping data of all 3 SNPs did not deviate from the Hardy–Weinberg equilibrium in the control group (*P*>0.05). In the discovery sample, only rs1460922^G^ was significantly associated with the risk of VT (*P*_adj_=0.015; OR: 1.54 [95% CI, 1.09–2.19]; Table 3) after adjusting for covariates of sex and age. Genotypic association analysis was then performed under different inheritance models (additive, dominant, or recessive). SNP rs1460922^G^ was significantly associated with the risk of VT in the additive model after adjusting for covariates of sex and age (*P*_adj_=0.010; OR: 1.64 [95% CI, 1.12–2.39]; Table 4) in genotypic association. SNPs rs4687326 and rs2686464 in *FGF12*, which failed to show significant association with risk of VT in the discovery sample (the adjusted *P* value is 0.390 for rs2686464 and 0.414 for rs4687326; Table 3), were excluded from the replication stage of the study.

**Table 3:** Analysis of Allelic Association of SNPs in FGF12B With VT/VF

SNP	Sample Size, n (Case/Control)	R.A	Frequency (Case/Control)	Without Adjustment		With Adjustment	
				*P*_obs_	OR (95% CI)	*P*_adj_	OR (95% CI)
rs2686464, Discovery sample	255/289	C	0.763/0.750	0.701	1.07 (0.76–1.51)	0.390	1.18 (0.81–1.71)
rs4687326, Discovery sample	255/289	T	0.230/0.217	0.637	1.08 (0.79–1.47)	0.414	1.14 (0.83–1.58)
rs1460922, Discovery sample	255/289	G	0.350/0.252	5.77E‐03	1.60 (1.14–2.23)	0.015	1.54 (1.09–2.19)
rs1460922, Replication sample	180/288	G	0.298/0.225	0.029	1.46 (1.04–2.05)	0.018	1.64 (1.09–2.48)
rs1460922, Combined sample	435/577	G	0.327/0.242	2.12E‐04	1.56 (1.23–1.98)	2.52E‐04	1.59 (1.24–2.03)

CI indicates confidential interval; OR, odds ratio; *P*_adj_, *P* value for association after adjusting for covariates of sex and age by multiple logistic regression analysis using SPSS version 17.0; *P*_obs_, *P* value for association before adjusting for covariates of age and sex by 2×2 contingence tables using PLINK version 1.07; R.A, risk allele; SNP, single‐nucleotide polymorphism; VF indicates ventricular fibrillation; VT, ventricular tachycardia.

**Table 4:** Analysis of Genotypic Association of SNPs in FGF12B Under 3 Genetic Models

SNP	Model	Without Adjustment		With Adjustment	
		*P*_obs_	OR (95% CI)	*P*_adj_	OR (95% CI)
rs2686464, Discovery sample	Dominant (C)	0.073	2.36 (0.90–6.18)	0.082	2.47 (0.89–6.84)
	Recessive (C)	0.682	0.92 (0.60–1.40)	0.848	1.05 (0.66–1.65)
	Additive (C)	0.122	···	0.392	1.18 (0.81–1.71)
rs4687326, Discovery sample	Dominant (T)	0.364	1.19 (0.82–1.73)	0.224	1.28 (0.86–1.89)
	Recessive (T)	0.447	0.72 (0.30–1.69)	0.606	0.79 (0.33–1.91)
	Additive (T)	0.377	···	0.414	1.14 (0.83–1.58)
rs1460922, Discovery sample	Dominant (G)	0.018	1.68 (1.09–2.58)	0.042	1.60 (1.02–2.51)
	Recessive (G)	0.017	3.18 (1.17–8.64)	0.030	3.17 (1.12–9.01)
	Additive (G)	0.011	···	0.010	1.64 (1.12–2.39)
rs1460922, Replication sample	Dominant (G)	0.190	1.33 (0.87–2.03)	0.104	1.53 (0.92–2.56)
	Recessive (G)	0.007	3.23 (1.32–7.90)	0.008	4.60 (1.48–14.29)
	Additive (G)	0.024	···	0.017	1.67 (1.10–2.56)
rs1460922, Combined sample	Dominant (G)	4.00E‐03	1.55 (1.15–2.08)	4.00E‐03	1.59 (1.12–2.17)
	Recessive (G)	3.70E‐04	3.16 (1.63–6.14)	1.00E‐03	3.31 (1.65–6.64)
	Additive (G)	2.79E‐04	···	1.79E‐04	1.64 (1.27–2.12)

CI indicates confidential interval; OR, odds ratio; *P*_adj_, *P* value for association after adjusting for covariates of sex and age by multiple logistic regression analysis using SPSS version 17.0; *P*_obs_, *P* value for association before adjusting for covariates of age and sex by 2×2 contingence tables using PLINK version 1.07.

We verified the association between rs1460922 and VT in an independent sample. The results showed that rs1460922^G^ was still significantly associated with VT (*P*_adj_=0.018; OR: 1.64 [95% CI, 1.09–2.48]; Table 3) after adjusting for covariates of sex and age. In genotypic association, rs1460922 was significantly associated with VT in recessive and additive models (recessive: *P*_adj_=0.008; OR: 4.60 [95% CI, 1.48–14.29]; additive: *P*_adj_=0.017; OR: 1.67 [95% CI, 1.10–2.56]; Table 4). In the combined sample of the 2 Chinese cohorts, the VT association remained significant for rs1460922^G^ (*P*_adj_=2.52E‐04; OR: 1.59 [95% CI, 1.24–2.03]). Significant genotypic association was also found assuming an additive model (dominant: *P*_adj_=4.00E‐03; OR: 1.59 [95% CI, 1.12–2.17]; recessive: *P*_adj_=1.00E‐03; OR: 3.31 [95% CI, 1.65–6.64]; additive: *P*_adj_=1.79E‐04; OR: 1.64 [95% CI, 1.27–2.12]; Table 4).

### Functional Variations of *FGF12* Identified in Patients With idopathic VT

The SNP rs1460922 was noted to be associated with VT in 2 independent case–control studies. Consequently, to further verify the new mutation of *FGF12* associated with the risk of VT, all exons of *FGF12* in 320 unrelated samples with idopathic VT showing a clear subtype of VT (including 31 patients with idopathic VT from discovery and replication samples) were resequenced.

**Figure 1:**
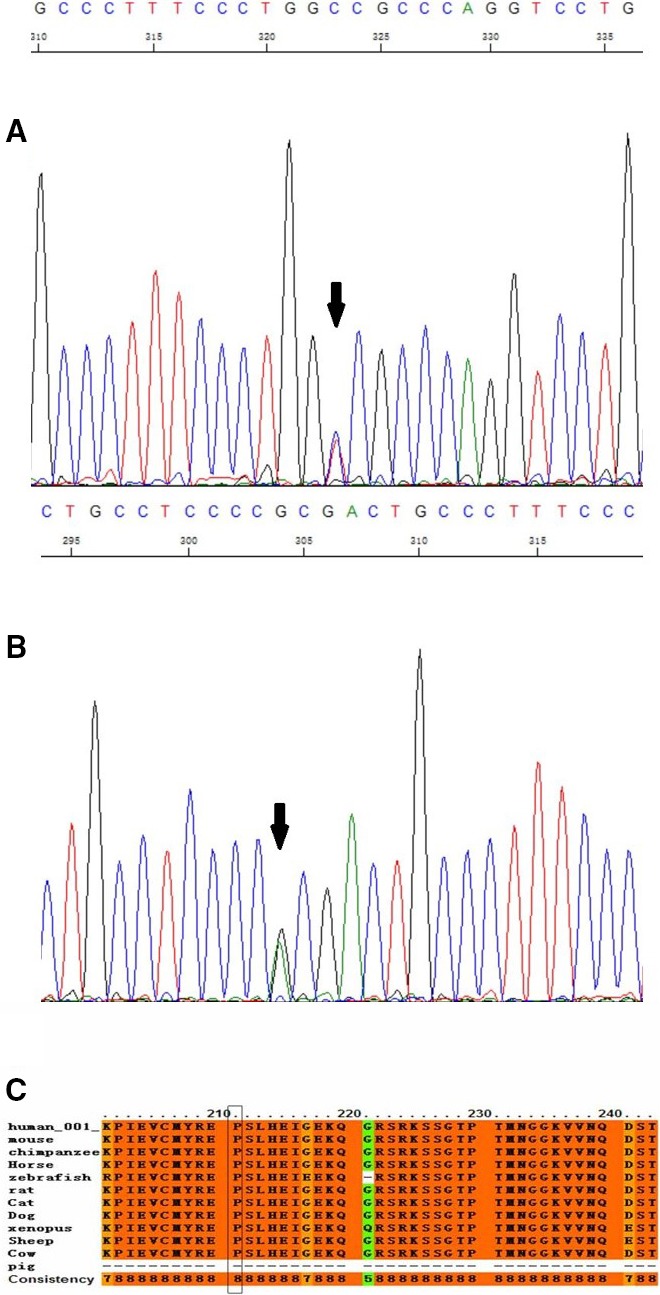
The sequencing results for variations and the conservation of 1 nonsynonymous variant and 1 functional SNP. A, The sequencing results for noncoding variation c.742C>T. B, The sequencing results for noncoding variation c.723G>A. C, The conservation of 1 nonsynonymous SNP rs17852067 (p.P211Q).

A nonsynonymous variation, rs17852067 (p.P211Q), in exon 5 of *FGF12* was identified in 3 (1%) participants with RVOT. Because no minor allele frequency (MAF) of rs17852067 was observed in National Center for Biotechnology Information (NCBI), ExAC, and 1000 Genomes databases, and the variation did not exist in 1000 controls in the present study, it is supposed that rs17852067 is a rare variation (MAF <0.01)^24^ associated with the disease. Two other rare variations (c.742C>T and c.723G>A in 2 different patients, respectively) in the 5′ UTR of *FGF12* were identified in 320 individuals with idopathic VT (Figure 1A and 1B, Table 5). Information of patients who carried these variations is shown in Table 5. Three common SNPs (MAF >0.01)^24^—rs3109189 (MAF of 0.0797 in our study and 0.1860 in NCBI), rs75224764 (MAF of 0.0010 in our study and 0.0016 in NCBI), and rs13088552 (MAF of 0.2953 in our study and 0.3372 in NCBI)—were identified in patients. In addition, the MAFs of these 3 SNPs identified in 320 patients with idopathic VT in this study appeared to be slightly different from the NCBI MAFs. It could be assumed that the NCBI MAFs are for the general population; if so, these SNPs may be associated with VT in the general population.

**Table 5:** The Clinical Information of VT Patients With Variations

GeneID	Mutation	Sex	Age, y	Diagnosis
634987	c.742C>T	Female	39	LOVT
529139	c.723G>A	Female	53	RVOT
614250	P211Q	Female	47	RVOT
633085	P211Q	Male	14	RVOT
662250	P211Q	Male	64	ROVT

LOVT indicates idiopathic ventricular tachycardia from the left ventricular outflow tract; RVOT, right ventricular outflow tract ventricular tachycardia; VT, ventricular tachycardia.

**Figure 2:**
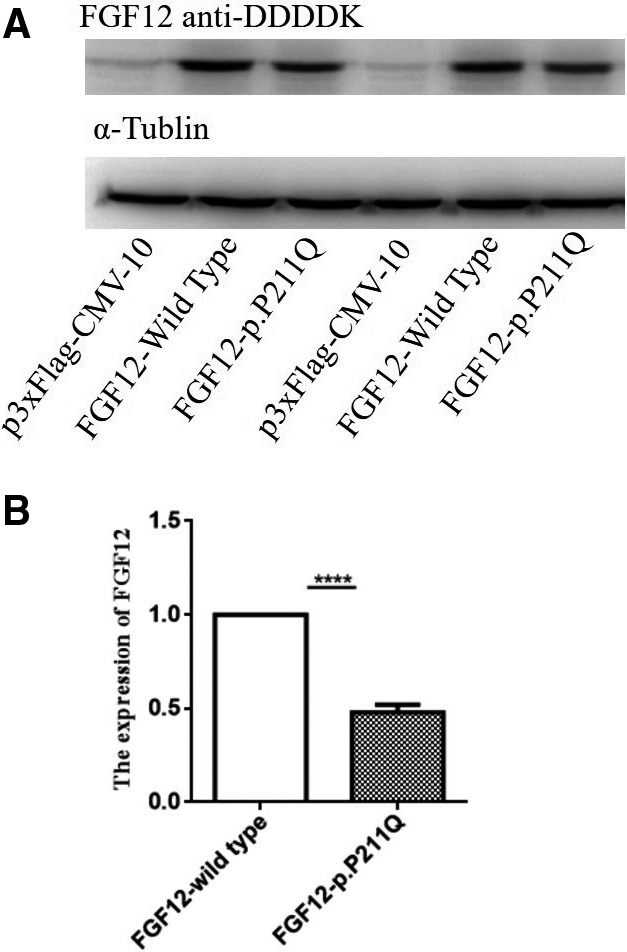
P211Q negatively regulates expression of FGF12 protein. A, P211Q significantly decreased the expression level of FGF12 protein in rat cardiac myocyte H9C2 cells compared with wild type by western blot analysis. α‐Tublin was used as loading control. B, The images of western blot analysis shown in (A) were scanned, quantified, and plotted. ****P <0.0001.

The p.P211Q mutation was highly conservative in most species (score: 8; Figure 1C). The prediction analysis results showed that p.P211Q was deleterious (Table 6). The score of 3 online programs predicting the injury of mutations was from 1 to 10. Lower scores showed higher injury in SIFT and Condel, and higher scores showed higher injury in PolyPhen2. Moreover, p.P211Q was noted to be highly deleterious in the 3 scoring programs (SIFT: 0.04 [deleterious]; PolyPhen2: 1 [probably damaging]; Condel: 0.849 [deleterious]). The regulation of p.P211Q at the protein level was also examined by western blotting. Protein extracts were isolated from rat myocardial H9C2 cells transfected with p3xFLAG‐CMV‐10‐FGF12‐Wt or p3xflag‐cmv‐10‐FGF12‐p.P211Q. The results of western blotting showed that the mutation p.P211Q significantly reduced 52% of the FGF12 protein expression (*P*<0.0001; Figure 2A and 2B).

**Table 6:** Critical Analysis Information of Variable Locus in *FGF12B*

Gene	Location	Type	Transcript	Mutation	SIFT	PolyPhen2	Condel
*FGF12B*	5′ UTR	Noncoding	ENSP00000413496	c.742C>T	···	···	···
	5′ UTR	Noncoding	ENSP00000413496	c.723G>A	···	···	···
	Exon	SNP	ENSP00000413496	rs17852067 (p.P211Q)	0.04 (deleterious)	1 (probably damaging)	0.849 (deleterious)

SIFT predicts whether an amino acid substitution affects protein function. SIFT can be applied to naturally occurring nonsynonymous polymorphisms or laboratory‐induced missense mutations. PolyPhen2 predicts the effect of an amino acid substitution on the structure and function of a protein. Condel is a general method for calculating a consensus prediction from the output of tools designed to predict the effect of an amino acid substitution. The Condel score is the consensus probability that a substitution is deleterious, so values nearer 1 are predicted with greater confidence to affect protein function. Chr indicates chromosome; SNP, single‐nucleotide polymorphism; UTR, untranslated region.

**Figure 3:**
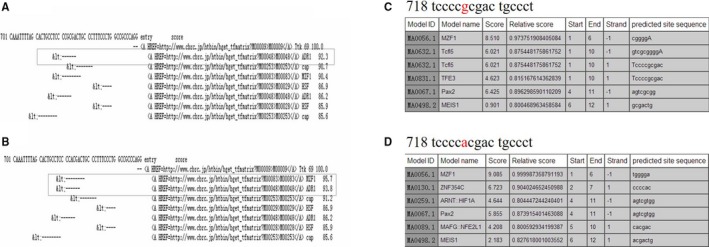
The prediction of binding domains for the transcription factors in the noncoding variation c.723G>A. A, The binding domains for the transcription factors before the gene point change predicted by TFSEARCH. B, The binding domains for the transcription factors after the gene point change predicted by TFSEARCH. C, The binding domains for the transcription factors before the gene point change by predicted JASPAR. D, The binding domains for the transcription factors after the gene point change predicted by JASPAR.

The prediction of binding domains for the transcription factors in the 2 mutation regions was noted. In case of c.723G>A, 2 transcription factors—MZF1 (myeloid zinc finger 1) and ZNF354C (zinc finger protein 354C)—were predicted to interact with the binding domain by TFSEARCH and JASPAR, respectively (Figure 3). In case of C.742C>T, no transcription factor was predicted to bind to the domain (data not shown). Based on the results of bioinformatics analysis, we cloned the region that contained the predicted MZF1 and ZNF354C binding sites and flanking sequences at the 5′ UTR of *FGF12* into the pGL3‐Basic‐REPORT luciferase vector, resulting in a reporter gene pGL3‐Basic‐FGF12‐WT with the G allele and pGL3‐Basic‐FGF12‐5′UTR‐Mut with the A allele. Each reporter was cotransfected with MZF1 or ZNF354c, and negative NC‐control (pcDNA3.1(+) or p3x flag‐CMV‐10 plasmid) into cells and luciferase assays was carried out. The results showed that the luciferase activities were not regulated by MZF1 and ZNF354c (Figure S3A and S3B).

## Discussion

In the present study, we observed that SNP rs1460922 of *FGF12* was associated with the risk of VT in the Chinese population. Furthermore, we identified a de novo functional variation (p.P211Q) that affects the expression of *FGF12* in idopathic VT (RVOT). To the best of our knowledge, this is the first time that the genetic association between *FGF12* and VT was observed.

We used 2 different approaches to test the association between *FGF12* and VT. First, we carried out a case–control association study with SNPs in *FGF12*. Significant association was demonstrated for the minor allele G of rs1460922 and VT after adjusting for covariates of sex and age (*P*_adj_=2.52E‐04; OR: 1.59 [95% CI, 1.24–2.03]; Table 3). The genotypic association was also significantly associated with VT in dominant, recessive, and additive models (dominant: *P*_adj_=0.004; OR: 1.59 [95% CI, 1.12–2.17]; recessive: *P*_adj_=0.001; OR: 3.31 [95% CI, 1.65–6.64]; additive: *P*_adj_=1.79E‐04, OR: 1.64 [95% CI, 1.27–2.12]; Table 4). These results indicated that common SNPs in *FGF12* can increase the genetic risk of VT.

Second, identification of de novo functional variations in patients with VT is important for confirming the association between *FGF12* and VT. Consequently, we resequenced all exons and exon–intron boundaries and 5′ and 3′ UTRs of *FGF12* in 320 patients with idopathic VT (idopathic VT is a special phenotype of VT in which patients represent only VT or premature ventricular contraction with normal structure of the heart^1^). According to the origin of idopathic VT, it is commonly classified into 3 types—idopathic VT from left ventricular outflow tract, idopathic VT from RVOT, and fascicular idopathic VT.^25^ RVOT is the most common form, accounting for 70% of all cases^26^ and 51% (163 cases with RVOT in 320 cases with idopathic VT) in our study. Five rare variations were identified after resequencing. A nonsynonymous variation, rs17852067 (p.P211Q), in exon 5 of *FGF12* was identified in 3 participants with RVOT (1% of all patients with idopathic VT, 2% of 163 patients with RVOT). The results of western blotting revealed that p.P211Q significantly reduced *FGF12* expression (52%, *P*<0.0001). Recent studies showed that some patients with BrS experienced RVOT leading to sudden death.^27^ idopathic VT is also a clinical symptom of BrS with ECG patterns similar to those of a left bundle‐branch block. Dysfunction of *SCN5A* is a major cause of both BrS and idopathic VT.^5^, ^28^, ^29^ These results are consistent with our study. A previous study^14^ reported that a variation in *SCN5A* (p.H1849R) could block the regulation of *FGF12* and cause human arrhythmia. In the present study, p.P211Q downregulated the expression of *FGF12* and might reduce binding to the Na_V_1.5 C‐terminus and Na^+^ channel current density, leading to an Na^+^ channel loss‐of‐function phenotype. The exact mechanism should be confirmed by further studies. In the present study, we could not detect more common variations (MAF >0.0001) because the sample size was limited.

It is interesting to note that FGF12 protein can interact with ion channels in the nervous and cardiac systems, bind to and modulate the cardiac Na_V_1.5 Na^+^ channel, and play a role in various arrhythmias, including VT.^6^, ^30^, ^31^, ^32^, ^33^ After binding to FGF12, recombinant Na_V_1.5 in human embryonic kidney 293 (HEK293) cells was observed to be a significant hyperpolarizing shift in the channel inactivation.^34^ Mutations of *FGF12*, such as p.P149Q, which decreased the binding affinity to the C‐terminus of specific voltage‐gated Na^+^ channels, affected the function of Na_V_1.5^33^ and induced cardiac arrhythmias. Another mutation of *FGF12*, p.Q7R, reduced *FGF12* expression and Na^+^ channel density and availability, leading to the development of BrS.^12^, ^13^ In contrast, abnormal mutations of genes encoding the Na^+^ channel, such as p.H1849R in *SCN5A*, can block the interaction and regulation of FGF12 and cause human arrhythmia.^14^ These studies suggested that the interaction of FGF12 and Na^+^ channels may play an important role in causing arrhythmia.

In conclusion, for the first time, we demonstrated a significant association between *FGF12* and VT and identified a de novo functional variation, p.P211Q (2% of 163 patients with RVOT), that can significantly downregulate *FGF12* expression. The exact mechanism underlying the development of VT/idopathic VT due to *FGF12* needs further validation and functional study.

## Sources of Funding

This work was supported by grants from National Basic Research Program of China (973 Program: 2013CB531103 and 2013CB531101), the National Natural Science Foundation of China (No. 91439109, 81270163, 81670363, 81630002, 31430047, and 91439129), NIH/NHLBI (USA) grants R01 HL121358 and R01 HL126729, Hubei Province Natural Science Programs (2016CFB224 and 2014CFA074), and the Program for New Century Excellent Talents at Chinese Universities (NCET‐11‐0181).

## Disclosures

None.

## Supporting information


**Figure S1.** Linkage disequilibrium structure and haplotype block in *FGF12B* in a Chinese Han population. Linkage disequilibrium (D′) for single‐nucleotide polymorphisms spanning a 266.3‐kb genomic region in *FGF12B* on chromosome 3 is generated by Haploview 4.0.
**Figure S2.** A schematic drawing giving the standard, stylized intron/exon gene map of *FGF12*. Three‐tag single‐nucleotide polymorphisms (SNPs) and area covered by the disequilibrium blocks they tag, the 3 new rare mutations, and the 3 common SNPs identified in patients by resequencing were indicated in the schematic drawing.
**Figure S3.** Transcription factors MZF1 and ZNF354C do not regulate the expression of the *FGF12* at the mutant site c.G723A. A, Effect of MZF1 on the pGL3‐Basic‐FGF12B‐5′UTRMut luciferase reporters compared with pGL3‐Basic‐FGF12B‐5′UTR‐Wt transfected into Hela cells. B, Effects of ZNF354c on the pGL3‐Basic‐FGF12B‐5′UTR‐Mut luciferase reporters compared with pGL3‐Basic‐FGF12B‐5′UTR‐Wt. Luciferase activities were calculated as the ratio of firefly/Renilla activities and normalized to the negative control (empty vectors) group. Results were obtained from 3 independent experiments. Data are shown as mean±SD.
