# Supplementary material for: De Novo FGF12 (Fibroblast Growth Factor 12) Functional Variation Is Potentially Associated With Idiopathic Ventricular Tachycardia
Source: J Am Heart Assoc. 2017 Aug 3;6(8):e006130. doi: 10.1161/JAHA.117.006130 (PMC5586455; doi:10.1161/JAHA.117.006130)
Supplement: Supplementary file 1 — Figure S1. Linkage disequilibrium structure and haplotype block in FGF12B in a Chinese Han population. Linkage disequilibrium (D′) for single‐nucleotide polymorphisms spanning a 266.3‐kb genomic region in FGF12B on chromosome 3 is generated by Haploview 4.0. Figure S2. A schematic drawing giving the standard, stylized intron/exon gene map of FGF12. Three‐tag single‐nucleotide polymorphisms (SNPs) and area covered by the disequilibrium blocks they tag, the 3 new rare mutations, and the 3 common SNPs identified in patients by resequencing were indicated in the schematic drawing. Figure S3. Transcription factors MZF1 and ZNF354C do not regulate the expression of the FGF12 at the mutant site c.G723A. A, Effect of MZF1 on the pGL3‐Basic‐FGF12B‐5′UTRMut luciferase reporters compared with pGL3‐Basic‐FGF12B‐5′UTR‐Wt transfected into Hela cells. B, Effects of ZNF354c on the pGL3‐Basic‐FGF12B‐5′UTR‐Mut luciferase reporters compared with pGL3‐Basic‐FGF12B‐5′UTR‐Wt. Luciferase activities were calculated as the ratio of firefly/Renilla activities and normalized to the negative control (empty vectors) group. Results were obtained from 3 independent experiments. Data are shown as mean±SD. [file JAH3-6-e006130-s001.pdf]

SUPPLEMENTAL MATERIAL

Figure S1. LD structure and haplotype block in *FGF12B* in Chinese Han population. Linkage disequilibrium (D') for SNPs spanning a 266.3kbps genomic region in *FGF12B* on chromosome 3, are generated by Haploview 4.0.

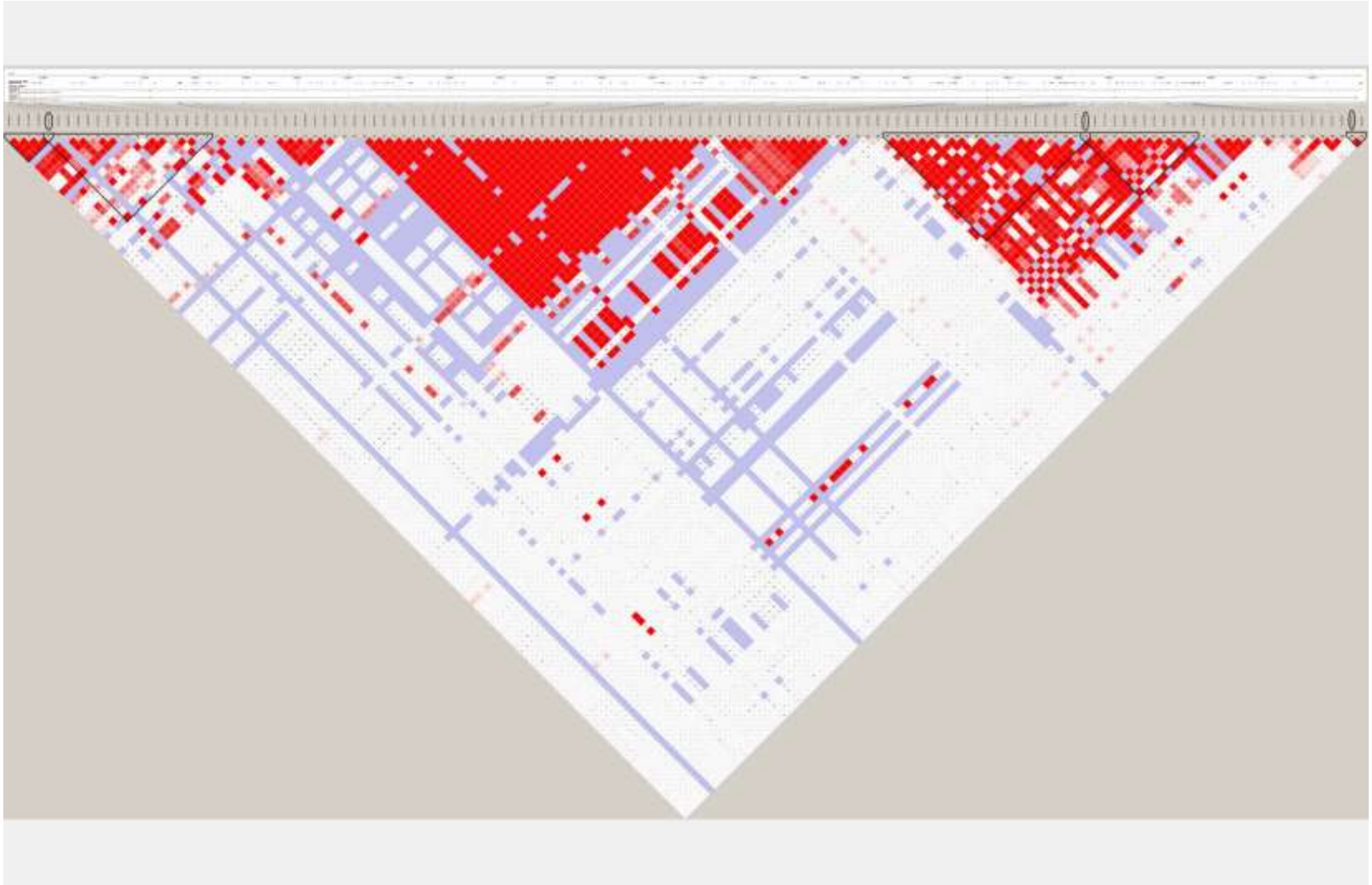

Figure S2. A schematic giving the standard, stylized intron/exon gene map of FGF12. Three tag SNPs and area covered by the disequilibrium blocks they tag, the three new rare mutations, and the three common SNPs identified in patients by the re-sequencing were indicated on the schematic.

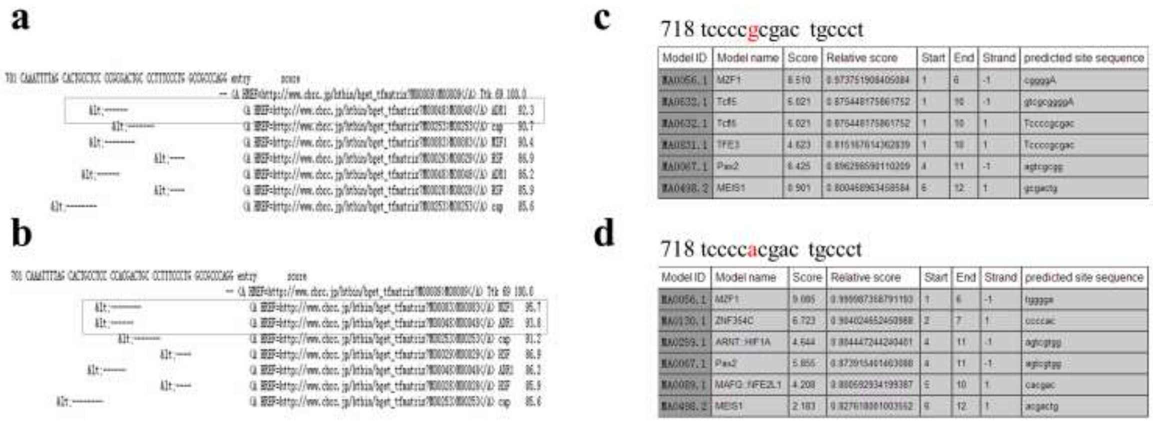

Figure S3. Transcription Factor MZF1 and ZNF354C do not regulate the expression of the FGF12 at the mutant site c.G723A. **a**, Effect of MZF1 on the pGL3-Basic-FGF12B-5’UTR-Mut luciferase reporters compared with pGL3-Basic-FGF12B-5’UTR-Wt transfected into Hela cells. **b**, Effects of ZNF354c on the pGL3-Basic-FGF12B-5’UTR-Mut luciferase reporters compared with pGL3-Basic-FGF12B-5’UTR-Wt. Luciferase activities were calculated as the ratio of firefly/renilla activities and normalized to the negative control (empty-vectors) group. Results were obtained from three independent experiments. Data are shown as means±SD.

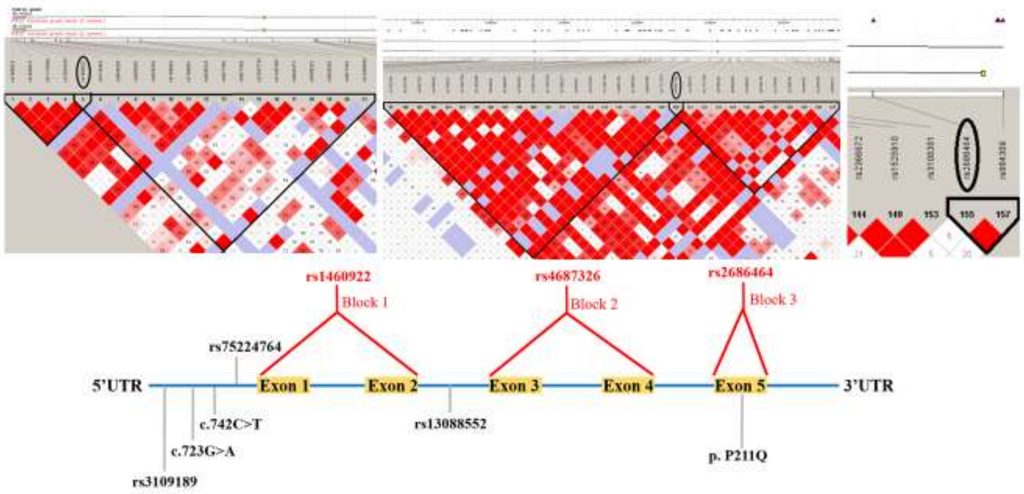

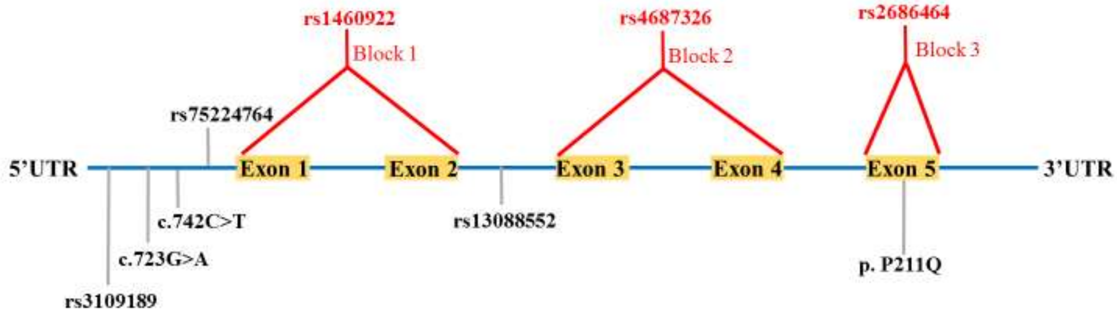
rs1460922
Block 1
rs4687326
Block 2
rs2686464
Block 3
rs75224764
5'UTR
Exon 1
Exon 2
Exon 3
Exon 4
Exon 5
3'UTR
c.742C>T
rs13088552
c.723G>A
p. P211Q
rs3109189
